# Mandibular Thickness Measurements as Predictive Tool for Specific Dental Disorders in Alpacas (Vicugna Pacos)

**DOI:** 10.3389/fvets.2022.817050

**Published:** 2022-02-24

**Authors:** Kirsten Proost, Bart Pardon, Lieven Vlaminck

**Affiliations:** ^1^Department of Surgery and Anesthesiology of Domestic Animals, Faculty of Veterinary Medicine, Ghent University, Ghent, Belgium; ^2^Department of Internal Medicine, Reproduction and Population Medicine, Faculty of Veterinary Medicine, Ghent University, Ghent, Belgium

**Keywords:** apical infection, periodontal disease, new world camelids, mandibular swelling, tooth root abscess, diastema, dental disease

## Abstract

**Background:**

Oral health in alpacas is often neglected until severe dental disease becomes evident under the form of a pronounced mandibular swelling with or without fistulation. Mandibular thickness measurements might serve as an easy tool to screen for early increases in thickness thereby identifying alpacas which could benefit from an oral examination.

**Objective:**

To study specific risk factors, including age, gender, level of performed measurements and dental disorders, associated with mandibular thickness in alpacas. To determine suitable cutoff values for mandibular thickness at specific locations for the development of a diagnostic test to identify animals with dental disorders.

**Study design:**

Cross-sectional study.

**Methods:**

Mandibular thickness was measured at standardized locations in 216 alpacas using a Vernier caliper. Risk factors for an increased mandibular thickness were collected through oral examination in sedated animals and by interview of the respective owner. A multivariable linear mixed model was built to identify factors associated with mandibular thickness. Cutoff values for specific dental disorders were obtained after receiver operating characteristics (ROC)-curve analysis.

**Results:**

Mandibular thickness was 0.43 ± Standard Error 0.21 mm [95% Confidence interval (CI) = 0.02-0.84 mm] higher at locations where interproximal gum recession was present (*P* = 0.039). Severe dental disease caused an increase in thickness of 1.90 mm (95% CI= 1.29-2.51 mm) (*P* < 0.001). Mandibular thickness with a cutoff of 19.4 mm showed a high accuracy for predicting severe dental disease (Se = 0.41; Sp = 0.92). Specifically, thickness at a level perpendicular to the medial canthus of the eye proved a more precise predictor for severe dental disease (AUC, 0.85; 95% CI 0.74-0.96; *P* < 0.001) with a lower cutoff of 18.5 mm and Sn and Sp of 0.52 and 0.82, respectively.

**Main limitations:**

No radiographic or computed tomographic studies were available to support the diagnosis of dental and/or apical disease.

**Conclusion:**

Mandibular thickness measurements in alpacas can aid early detection of animals in need of specialized dental care. Most animals with an increased mandibular thickness suffer from advanced dental disease. However, routine dental examinations remain necessary to allow the early detection of dental disorders in alpacas.

## Introduction

Dental disease is increasingly recognized as an important problem in the domesticated alpaca population (1, 2). Among the different dental diseases, tooth root abscesses, recently termed “apical infections,” are most frequently described and the predominant reason for medical intervention (2–5). Up until recently, only this advanced disease process was acknowledged and received attention as an important medical problem since oral examinations are not yet routinely performed in this species (1). A localized mandibular swelling is generally accepted to be a clinical sign most likely associated with apical disease in hypsodont species (2, 6, 7). Besides apical disease, the differential diagnosis for a swelling at the level of the mandible can include the packing of partially masticated food the mouth, a soft tissue abscess, a salivary mucocele, external trauma with or without a fissure or fracture of the mandible, and neoplasia (2). Recent studies in which thorough oral examinations have been performed in sedated animals using a dental mirror or a portable oroscope provide novel insights in the true prevalence of specific dental disorders in alpacas (1). Common dental disorders readily diagnosed included diastemata (43.1%), wear abnormalities (39.6%) and periodontal disease (PD) (33.3%) in a large subset of animals, providing evidence to conclude that apical disease should be considered an advanced stage of dental disease often preceded by less severe and potentially treatable dental disorders (1). In the same field study, subjectively palpable mandibular swellings were associated with PD (8). Dimensions of mandibular arcades may be an easily accessible straightforward tool for veterinarians and owners to predict the presence of specific dental disorders in an individual alpaca, allowing a timelier identification of diseased animals which would benefit from an oral examination. Evaluation of mandibular swelling is however subjective, and there is a need for advice on measurement and cut-off values to standardize diagnosis. Currently, scientific knowledge regarding the normal and pathological thickness of the mandibular arcade at different levels in alpacas is lacking. Age and gender effects are thought to possibly influence mandibular thickness in the growing skull. Therefore, the first objective of the current study was to study the association of specific risk factors, including age, gender, level, and dental disorders, with the mandibular thickness in alpacas. The second was to determine suitable cutoff values for mandibular thickness at specific locations in function of the development of a diagnostic test to identify at-risk animals and to predict specific dental disorders.

## Materials and Methods

### Study Design

A cross-sectional study was conducted on 24 alpaca farms located in the Northern part of Belgium and the Southern part of the Netherlands. Participating alpaca farms were selected out of 33 (response rate = 15.3%) interested farms, after a call addressing the members of the Alpaca Association Benelux (*n* = 215 herds). Selection of participating farms was performed conveniently, based on traveling distance for the primary investigators. The data used for this study was collected in a large-scale study aiming to determine the prevalence of cheek teeth disorders in alpacas. The number and percentage of animals at each farm to be examined was determined in cooperation with the owner depending on their economic value and gestational status of the female animals. The acquired sample size (*n* = 216) allowed the detection of a 0.8 mm difference in mean mandibular thickness between the two test groups (diseased vs. not diseased) using a mean for the reference group of 16.8 mm and a standard deviation of 2 mm with 80% power and 95% confidence.

### Clinical Examination and Data Collection

**Figure 1:**
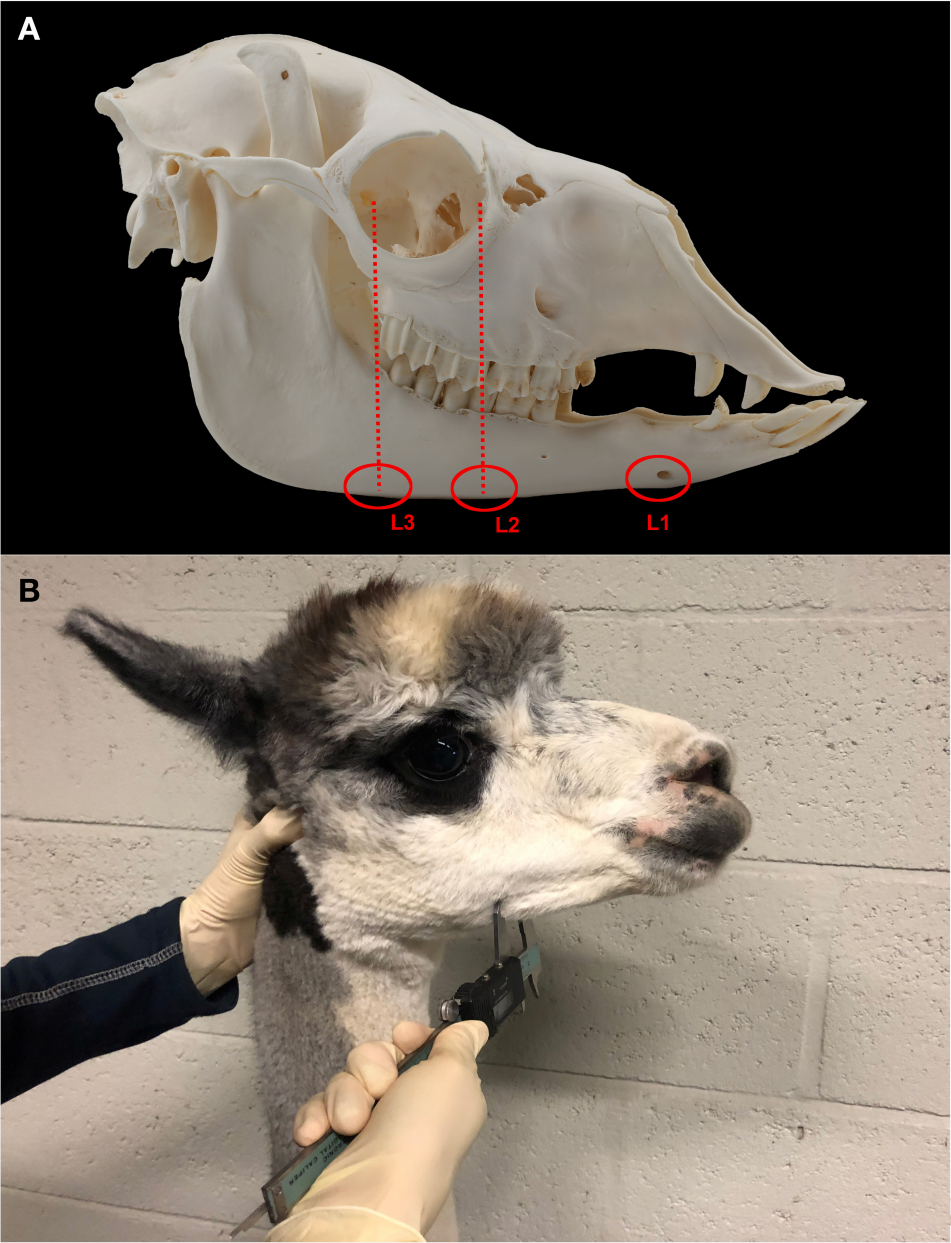
Location of performed measurements at the level of the mandibular arcade **(A)**. The mandibular thickness was measured at the level of the symphysis of the mandible (L1) in all included animals. Furthermore, the thickness of each hemimandible was measured (left and right) at two locations based on a line drawn from the mesial aspect of the medial canthus of the eye or from the lateral canthus of the eye, respectively, perpendicular to the longitudinal axis of each mandibular arcade. All measurements were performed using a digital vernier caliper as illustrated **(B)**.

Age of the examined animals at the time of the farm visit was derived from farm logs if possible. Furthermore, gender and neuter status of all investigated animals was recorded. Body condition was scored in each animal using a preset scoring system (9). Individual teeth were identified using the modified Triadan System (10). The mandibular thickness was defined as the thickness of the mandibular ramus measured at a specific location. Five measurements of the thickness of the mandibular arcades were performed in 216 animals using a Vernier caliper at predetermined locations (L1 = symphysis, L2L = left front, L3L = left back, L2R = right front, L3R = right back). These five locations were allocated to three levels (L1 = symphysis, L2 = Level 2, L3 = Level 3) (Figure 1). In the authors' experience, dental pathological changes involving abnormalities of the symphysis region are rare in this species. Given mandibular swellings are primarily reported as a hallmark sign for tooth root abscesses at the cheek teeth level (2), emphasis will be put on measurements of the mandibular width centered over the root structures of the mandibular cheek teeth. L2 corresponds to root structures of Triadan 09 and the mesial part of Triadan 10, whereas L3 corresponds with the root structures of the distal root of Triadan 10 and of Triadan 11. Oral examinations were performed under deep sedation acquired after administering ketamine (5 mg/kg IM, Ketamidor, Richter Pharma, Austria) and medetomidine (30 μg/kg IM, Sedator, Eurovet, Bladel, Belgium) or dexmedetomidine (15 μg/kg IM, Dexdomitor, Orion Corporation Orion Pharma, Espoo, Finland) intramuscularly. Food particles were removed from the oral cavity through extensive flushing. A miniature/pony speculum (Capps miniature/pony speculum, Capps Manufacturing Inc., Cortland, NE, United States) was used in combination with a dental speculum light (MiCH lite, MiCH, Mechelen, Belgium). All teeth and surrounding soft tissues were inspected using a dental mirror or a rigid portable dental endoscope (Karl Storz, Tuttlingen, Germany). Specific cheek teeth abnormalities including PD, diastemata (with or without the presence of periodontitis), pulpar exposure, displaced teeth, wear abnormalities and missing teeth were identified as previously described (1). Different clinical expressions that can be associated with periodontal disease such as gingival recession, drainage of purulent material and increased tooth mobility were individually recorded, and the severity of these changes were subjectively graded. All palpations, measurements and dental examinations were performed by the same two investigators (KP and LV).

### Statistical Analysis

**Figure 2:**
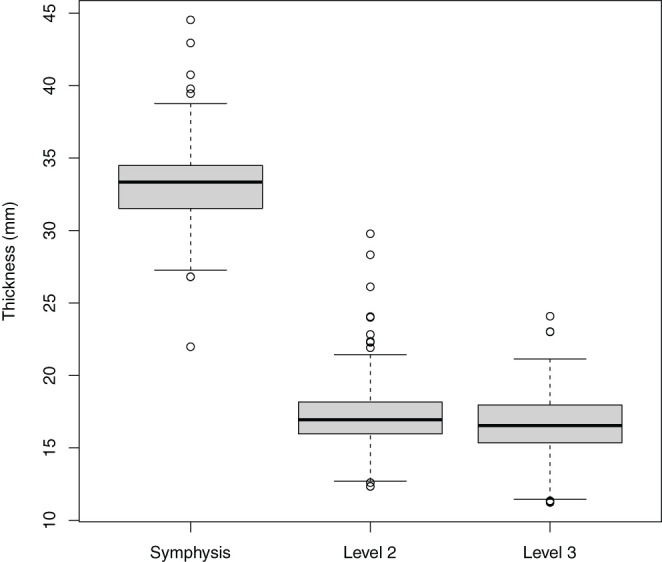
Boxplots illustrating the mandibular thickness according to measurement level as measured in 216 alpacas, thereby providing 1,080 measurements. Levels included Symphysis, Level 2, and Level 3.

The thickness of the mandibular arcade at a specific mandibular location was the outcome of interest. A multivariable mixed effects linear regression model (glmer) was built with mandibular thickness as outcome and alpaca nested within herd added as random factor to account for clustering. The data was split according to the specific mandibular level based on the analysis of descriptive statistics (Figure 2). Measurements of the mandibular thickness at L2 and L3 were retained for further analysis. To allow further statistical analysis of specific clinically relevant dental disorders with a low prevalence, the grouping variable “severe dental disease” was created. Cases were categorized in this variable in the presence of pulp exposure and/or purulent gingival drainage, and/or increased mobility of one or more teeth. These cases represent advanced disease processes such as endodontic disease, apical infection, and/or severe periodontal disease that would require specific veterinary intervention in a clinical setting.

First the association of each of the 14 risk factors with the outcome was tested by performing a univariable mixed effects linear regression analysis. Risk factors included age (<1.5 years/1.5-7 years/7.1-17 years), level (2/3), mandibular arcade (left/right), gender (female/male/male castrated) and specific dental disorders consisting of periodontal disease, interproximal gum recession, purulent drainage, increased mobility, diastemata, pulpar exposure, displaced teeth, wear abnormalities, missing teeth, and severe dental disease. Factors with *P* < 0.20 and sufficient observations (*n* > 30) were retained for the multivariable model. Correlations between significant risk factors were tested using the phi-coefficient and Cramér's V. When strongly, significantly correlated (>0.60), only the most clinically relevant variable was maintained in the model. Hereby, individual dental disorders were chosen over created “grouping variables.” In this way, diastemata and interproximal gum recession received priority for inclusion at the start of the model building process. The multivariable model was built stepwise backwards, gradually excluding non-significant variables. No significant biological interactions were identified between main effects. The deviance residuals were plotted against the predicted probabilities to inspect model adequacy. Furthermore, a half-normal probability plot was constructed to inspect model adequacy and potential outliers. A plot of the delta deviance statistics was used to check for evidence of potential influential observations. Model fit was determined using the Nakagawa R^2^ (11). Receiver operating characteristic (ROC) curve analysis was performed to determine the optimal cut-off for mandibular thickness in predicting specific dental disorders. The area under the curve, reported with 95% confidence intervals (CI) was used as a summary measure for determination of the diagnostic accuracy. Youden's index (Y = Sensitivity (Sn) + Specificity (Sp) −1), was used to determine cutoffs for each variable. Sn, Sp, and accuracy were determined. Significance was set at *P* < 0.05. R V4.1.0., R foundation for statistical analysis was used for statistical analyses.

## Results

### Descriptive Results

After visiting 24 alpaca farms, 216 animals were included in the study. The study population consisted of 211 Huacaya and 5 Suri alpacas. Mean number of animals originating from each farm was 9 ± 7 (range 1-28 animals). Average sampling rate was 38 ± 24 % (range 3-73%). The mean age of alpacas included in the study was 5.6 ± 3.2 years (range 0.8-16.3). The study population consisted of 105 females (62.1%), 49 intact males (19.3%), and 62 castrated males (18.7%). The body condition score of studied animals ranged from 0.5 to 4.5 with a mean of 2.5 ± 1.

A total of 1,080 mandibular thickness measurements were obtained. Mean L1 width (33.2 ± 2.8 mm, 216 measurements) differed strongly in comparison to mean L2 (17.1 ± 2.0 mm, 432 measurements) and L3 (16.6 ± 2.1 mm, 432 measurements) values, necessitating a split of the dataset to achieve normality (Figure 2). Further statistical analysis was performed using only the measurements obtained at level 2 and 3.

### Risk Factor Analysis

**Figure 3:**
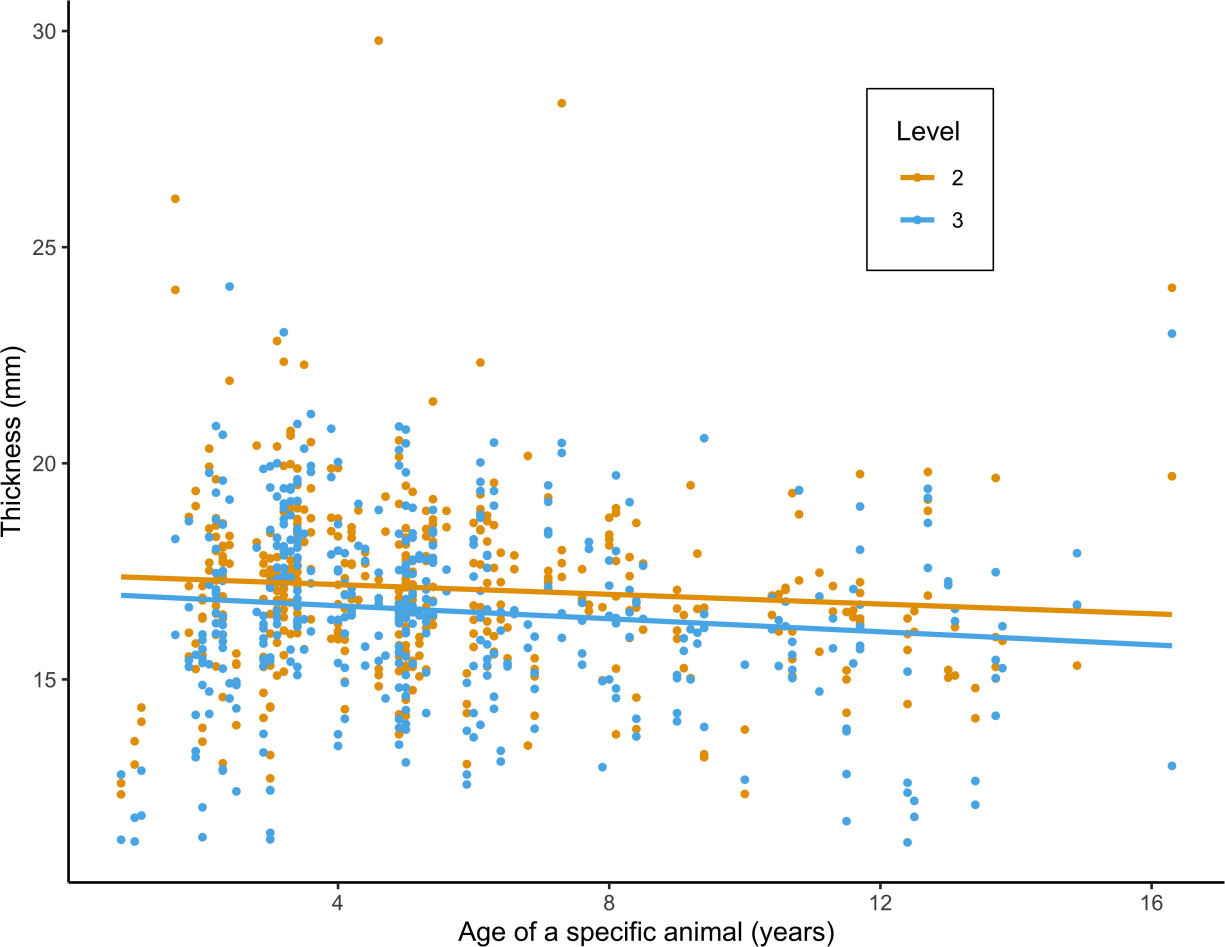
Scatterplot of mandibular thickness (mm) at level 2 (L2) and 3 (L3) in relation to age (years), as measured in 216 alpacas. Values and fitted regression lines of the 864 obtained measurements at L2 and L3 are depicted in orange and blue, respectively.

Univariable analysis identified nine variables as being significantly associated with increased mandibular thickness, including age category, level, PD, interproximal gum recession, drainage of purulent material, increased tooth mobility, diastema, pulp exposure, and severe dental disease proved statistically significant (Table 1). Only factors with >30 observations were retained for multivariable analysis. PD was significantly correlated with the presence of diastemata and interproximal gum recession. Only the presence of diastemata and interproximal gum recession were added at the start of the model building process since they were not mutually correlated. The final model consisted of four significant factors associated with L2 and L3 thickness (Table 2). The mandibular thickness is expected to be 3.61 mm (95% CI 1.85-5.40 mm, *P* < 0.001) and 3.01 mm (95% CI 1.23-4.82 mm, *P* < 0.001) higher in animals aged 1.5-7 years and 7.1-17 years compared to younger animals, respectively. Mandibular thickness is expected to be 0.46 mm (95% CI 0.27-0.64 mm) lower at L3 compared to L2 (*P* < 0.001) (Figure 3). Also, mandibular thickness is expected to be 0.43 mm (95% CI 0.02-0.84 mm) higher at locations where interproximal gum recession is present (*P* = 0.039). Furthermore, severe dental disease is expected to cause an increase in mandibular thickness of 1.90 mm (95% CI 1.29-2.51 mm) (*P* < 0.001). A calculated conditional R^2^ for the final multivariable model indicates 53.8% of variance in mandibular thickness can be predicted based on random effects comprising farm and animal, and all significantly associated fixed effects. Eleven percent of the variance in the mandibular thickness can be explained solely based on the detected statistically relevant fixed effects, including age category, level, interproximal gum recession and severe dental disease. A ROC analysis was performed for all variables proven significantly associated with mandibular thickness after performing the univariable analysis. The general and level specific results of performed ROC analysis are summarized in Table 3. In general, mandibular thickness shows a high accuracy for predicting severe dental disease (AUC, 0.70; 95% CI 0.60-0.80) with a cutoff of 19.4 mm. A low Sn and high Sp were found. Specifically, at L2, mandibular width showed an equally high accuracy (AUC, 0.70; 95% CI 0.57-0.83; *P* = 0.001) with a lower cutoff of 18.5 mm and Sn and Sp of 0.52 and 0.82, respectively.

**Table 1:** Mean mandibular thickness over different categorical variables at the level of the mandibular rami measured at four locations (L2, R2, L3, and R3).

		***n***	**%**		***n***	**%**		***n***	**%**	***P-*value**
	**<1.5 years**			**1.5-7 years**			**7.1-17 years**			
Age (categorical)	12.65 ± 1.00^ab^	12	1%	17.04 ± 1.98^a^	636	74%	16.48 ± 2.10^b^	216	25%	<0.001
	**2**			**3**						
Level	17.10 ± 2.01^a^	432	50%	16.58 ± 2.11^a^	432	50%				<0.001
	**Left**			**Right**						
Mandibular arcade	16.79 ± 1.95	432	50%	16.90 ± 2.19	432	50%				0.25
	**Female**			**Male**			**Male castrated**			
Gender	16.69 ± 2.08	420	49%	16.41 ± 2.14	196	23%	17.44 ± 1.87	248	29%	0.823
**Dental disorders**	**No**			**Yes**						
PD	16.76 ± 1.97^a^	760	88%	17.48 ± 2.63^a^	104	12%				<0.001
IGR	16.75 ± 1.95^a^	778	90%	17.69 ± 2.81^a^	86	10%				<0.001
Purulent drainage	16.81 ± 2.03^a^	854	99%	19.83 ± 3.43^a^	10	1%				<0.001
Increased mobility	16.80 ± 2.00^a^	852	99%	20.00 ± 4.05^a^	12	1%				<0.001
Diastamata	16.74 ± 1.96^a^	699	81%	17.29 ± 2.44^a^	165	19%				<0.001
Pulpar exposure	16.80 ± 2.00^a^	846	98%	18.78 ± 3.92^a^	18	2%				<0.001
Displaced teeth	16.83 ± 2.06	849	98%	17.59 ± 2.69	15	2%				0.977
Wear abnormalities	16.83 ± 2.09	743	86%	16.96 ± 2.00	121	14%				0.271
Missing teeth	16.85 ± 2.07	856	99%	16.74 ± 2.37	8	1%				0.82
Severe DD	16.75 ± 1.93^a^	830	96%	19.08 ± 3.63^a^	34	4%				<0.001

*Mean ± standard deviation for continuous data and number (n) or percent (%) for frequency data. PD, Periodontal disease; IGR, Interproximal gum recession; Severe DD, Severe dental disease consists of cases diagnosed with purulent gingival drainage and/or increased tooth mobility and/or pulp exposure. Variables (a, b) with the same superscript within the same row are statistically different at P < 0.001. A total of 864 measurements were obtained from 216 alpacas. Univariable mixed models have been used to generate the represented P-values. Alpaca nested within herd was added as a random factor*.

**Table 2:** Final multivariable mixed model showing factors associated with the thickness of the mandibular rami measured at level 2 and level 3 in 216 alpacas.

**Variable**	**Category**	***n***	**%**	**Estimate**	**SE**	**95% CI**	***P***
Intercept				13.40	0.90	11.62-15.16	<0.001
Age	<1.5 years	12	1.4	Referent			
	1.5-7 years	636	73.6	3.61	0.90	1.85-5.40	<0.001
	7.1-17 years	216	25.0	3.01	0.91	1.23-4.82	0.001
Level	2	432	50.0	Referent			
	3	432	50.0	−0.46	0.10	−0.64-0.27	<0.001
IGR	No	778	90.0	Referent			
	Yes	86	10.0	0.43	0.21	0.02-0.84	0.039
Severe dental disease	No	830	96.1	Referent			
	Yes	34	3.9	1.90	0.31	1.29-2.51	<0.001

*SE, Standard error; IGR, Interproximal gum recession*.

*Alpaca nested within herd was added as a random factor*.

**Table 3:** Results of receiver operating characteristic (ROC) analysis.

**General**								
**Variable**	**#pos**	**%pos**	**AUC**	**95% CI**	**Cut-off**	**Sn**	**Sp**	***P***
PD	104	12,0%	0.55	0.79-0.61	19.30	0.19	0.92	0.079
IGR	86	10,0%	0.58	0.51-0.65	18.93	0.27	0.89	0.015
Purulent drainage	10	1,2%	0.46	0.28-0.64	15.37	0.9	0.21	0.645
Increased mobility	12	1,4%	0.45	0.31-0.59	14.915	1	0.14	0.537
Diastemata	165	19,1%	0.50	0.45-0.54	17.635	0.35	0.68	0.848
Pulpar exposure	18	2,1%	0.62	0.49-0.74	16.105	0.89	0.34	0.090
Severe dental disease	34	3,9%	0.70	0.60-0.80	19.365	0.41	0.92	<0.001
**Level 2**								
**Variable**	**#pos**	**%pos**	**AUC**	**95% CI**	**Cut-off**	**Sn**	**Sp**	***P***
PD	61	14,1%	0.56	0.48-0.64	19.30	0.23	0.93	0.160
IGR	47	10,9%	0.60	0.51-0.70	19.30	0.30	0.93	0.048
Purulent drainage	6	1,4%	0.84	0.70-0.97	17.37	0.40	0.60	0.005
Increased mobility	10	2,3%	0.78	0.61-0.96	19.39	0.60	0.93	0.002
Diastemata	106	24,5%	0.53	0.47-0.60	16.11	0.82	0.32	0.287
Pulpar exposure	12	2,8%	0.60	0.41-0.78	18.71	0.42	0.84	0.261
Severe dental disease	23	5,3%	0.70	0.57-0.83	18.48	0.52	0.82	0.001
**Level 3**								
**Variable**	**#pos**	**%pos**	**AUC**	**95% CI**	**Cut-off**	**Sn**	**Sp**	***P***
PD	43	10,0%	0.54	0.45-0.63	14.98	0.91	0.20	0.416
IGR	39	9,0%	0.55	0.46-0.65	16.365	0.67	0.45	0.263
Purulent drainage	4	0,9%	0.67	0.35-0.99	19.79	0.50	0.94	0.234
Increased mobility	2	0,5%	0.58	0.037-1.00	20.53	0.5	0.98	0.685
Diastemata	59	13,7%	0.52	0.44-0.60	14.31	0.93	0.15	0.581
Pulpar exposure	6	1,4%	0.70	0.48-0.92	19.37	0.50	0.91	0.091
Severe dental disease	11	2,5%	0.68	0.51-0.85	19.37	0.46	0.91	0.043

*Optimal cutoffs of mandibular thickness in the prediction of different dental disorders over 864 measurements (General) or 432 measurements (Level 2 and 3) in 216 alpacas, respectively*.

*#pos, number of positive observations; %pos, percentage of positive observations; AUC, Area under the curve as measure for accuracy; CI, Confidence interval; Sn, Sensitivity; Sp, Specificity; PD, Periodontal disease; IGR, Interproximal gum recession; Severe dental disease, Severe dental disease consists of cases diagnosed with purulent gingival drainage and/or increased mobility and/or pulp exposure*.

## Discussion

Oral disease in alpacas often remain unnoticed until severe dental disease becomes evident under the form of mandibular swelling with or without fistulation, weight loss or other less common and non-specific clinical signs associated with dental pathological changes (1). The first aim of this study was to determine the influence of specific factors, including age, gender, and specific dental disorders on the mandibular thickness at specific locations in alpacas. The secondary goal was to yield an easily accessible field observation tool for the detection of specific animals in need of dental examinations. To the authors' knowledge, this is the first study looking at mandibular thickness in relation to age, gender, and specific dental disorders. This information could be of great help to identify animals with dental issues as part of a clinical examination. The majority of alpaca owners may be hesitant to have a thorough oral examination performed on their animals given the associated costs and often ungrounded and disproportionate expectations of risks associated with sedation. Also, the fact that dental care is not yet habituated in this species as is also the case in true ruminants, further complicates the situation. In contrast, in equine medicine, routine dental examinations in the sedated animal are readily accepted as a part of good veterinary practice. By performing dental check-ups on a regular base, disease processes can often be diagnosed and treated in an earlier stage, not associated with an increase in mandibular thickness, leading to a better overall prognosis. Treatment strategies dealing with minor abnormalities are less invasive compared to treatment strategies for advanced dental disease and have a better prognosis. Small corrections of wear, clearing impacted food from diastemata and periodontal pockets and occluding small diastemata, often provides easily accessible means to prevent the development of severe dental disease (1). In animals suffering from more advanced dental disease, exodontia is often the only remaining option to allow healing in these often chronic cases thereby guaranteeing quality of life for the animal (2). Given the rather low number of cheek teeth contributing to the occlusal surface in alpacas, care should be taken to limit the need for dental extractions.

As expected, mandibular thickness was found to be significantly smaller in younger animals, demonstrating the stage of development of the skull in this age group. Furthermore, mandibular thickness was shown to be significantly higher at L2 compared to L3, although the estimated difference is expected to be lower than 0.5 mm. Interestingly, a statistically significant increase in mandibular thickness could already be observed in the presence of interproximal gum recession, which is in line with the association found between subjectively palpable mandibular swellings and periodontal disease (8). Such thickening of the mandibular ramus is indicative for a pronounced inflammatory response at the level of the associated periodontal tissues. Further histological research is necessary to characterize the specific nature of these changes in mandibular thickness associated with interproximal gum recession. In the presence of severe dental disease, in this study characterized by pulpal exposure and/or purulent gingival tracts and/or increased tooth mobility, mandibular thickness is expected to increase more significantly, by almost 2 mm. This value reflects the more chronic nature of these conditions causing a more pronounced thickening of the mandibular ramus.

To allow the specific identification of animals possibly benefiting from oral examinations and further treatment, cutoff values for specific dental disorders were calculated. In this study it was concluded, if the mandibular thickness measured at location L2 or L3 in a specific animal would exceed 19 mm, a more thorough oral examination is warranted to identify the presence of possible important dental disorders. This parameter showed a high Sp although also a low Sn which would mean that measuring normal values of mandibular thickness does not exclude the presence of any dental disorders. This supports the hypothesis that measuring jaw thickness is not accurate enough to replace routine oral examinations. However, when a thickening of the mandibular arcade is present, oral examination is urgently needed (higher Sp). L2 mandibular thickness demonstrated a lower cutoff value of 18.5 mm in the presence of severe dental disease. A relatively high Sn and Sp indicate mandibular thickness at this specific level to be a good parameter to screen for advanced dental disease. Nevertheless, early-stage dental disease cannot be recognized in this way at this specific location. No cutoff values could be established for performing specific measurements at the L3 location which was attributed to the rather low number of positive observations at this specific level. Unfortunately, only a low number of animals younger than 1.5 years (*n* = 12) was included in the current study, hampering the development of age-group specific normal cutoff values. The importance of this lack of scientific data should however not be overestimated given the low prevalence of dental disorders in this age group as previously demonstrated (1).

Small deviations from the normal mandibular thickness appear difficult to assess in the day-to-day veterinary practice. In our opinion, differences lower than 1 mm will be difficult to perceive given we should take some observer or measurement bias into account. Also, not all animals will be easy to handle and will complicate successful measuring since alpacas are known to perceive stress when touching the head (12). Measurements of mandibular arcade thickness should be reserved for animals used to being handled or in which sufficient immobilization of the head can be guaranteed. Only measurements which can be performed in a controlled way are expected to be of value given the relatively small differences in thickness important to observe (Table 1). Measurements of the mandibular thickness also fail to provide information on the dental health status of the cheek teeth in the maxillary arcade. Despite the lower prevalence of dental disorders at the maxillary arcade, impact of chronic severe dental disorders on animal welfare should not be underestimated. Measurements of the mandibular thickness can be an easily accessible additional tool for alpaca owners to screen for animals in need of a dental check-up.

Despite the possible advantages of mandibular thickness measurements as a tool to assess the necessity for a more thorough dental exam, its value should not be overestimated. A better evolution in alpaca medicine might be if annual dental screenings would become common practice in alpaca health programs. A high prevalence of dental disorders further supports this theory. Recent studies have shown a prevalence of diastemata, wear abnormalities and periodontal disease at the level of the cheek teeth in 43.1, 39.6, and 33.3 % of examined animals, respectively (1). To allow efficient and reliable detection of oral and dental disorders, these oral examinations should only be performed in deeply sedated animals. Attempts to perform these examinations in unsedated animals will invariably lead to defensive behavior and extensive tongue movements (12). This will complicate oral examinations providing unreliable results and might even lead to dangerous situations for both animal and bystanders. Using the reported sedation protocol, the authors have not experienced any complication during the oral exams in any of the animals included in this study. It allowed a thorough oral examination in all animals with some requiring a top-up of one third of the initial dose. All animals recovered uneventfully. Leading us to question the objective risk associated with this required sedation. As already previously demonstrated, protocols using a combination of α-agonists and ketamine appear safe to use in camelids (13–15). However, owners seemed reluctant to have animals sedated because of expected risks for abortion in pregnant animals. Nevertheless, xylazine the most commonly used α2-agonist in veterinary practice in camelids, has been used extensively without associated abortions as there have been reported in cattle in the third trimester (16, 17). Owner education regarding specific risks associated with specific sedation protocols can be a future challenge for camelid vets to allow good dental preventative veterinary medicine.

Our study design was prone to several limitations. Misclassification bias is expected to be present given the limitation of the on farm clinical exam. To allow a reliable characterization of the perceived dental pathological changes, radiography and/or computed tomography remain(s) necessary in a subset of severely affected animals. The clinical diagnosis on the oral examination is only a presumptive diagnosis, especially in animals suffering from apical disease. Furthermore, selection bias cannot be ruled out since alpaca farms were selected based on traveling distance after a call spread to the Alpaca Association Benelux. Also, animals of exceptional high value or at an early or late gestation stage were most often withdrawn from the study based on the owner's consent to let specific animals participate.

In conclusion, an increase in mandibular thickness was significantly associated with increasing age (>1.5 years), interproximal gum recession and severe dental disease. The latter characterized by the presence of pulpal exposure and/or purulent gingival drainage and/or increased tooth mobility. In general, measurements of the mandibular thickness exceeding 19 and 19.4 mm were indicative of interproximal gum recession and severe dental disease, respectively. However, lower values than this cut-off should be interpretated carefully given the low Sn. More specifically, an objective measurement of mandibular width can be performed at a location determined by drawing an imaginary perpendicular line starting at the medial canthus of the cis mandibular arcade (L2). A measurement of >18.5 mm at this location suggested severe dental disease, with a low Sn and reasonable Sp, and diagnostic accuracy. Nevertheless, only animals affected by severe dental disorders can be detected when measuring the thickness of the mandibular arcade. The acquired information can aid veterinarians and alpaca owners to detect animals in urgent need of dental care. In general, routine oral examinations remain the best option to allow an early detection of dental disease, thereby facilitating successful treatments and favorable long-term prognosis.

## Author's Note

A request for participation was spread to alpaca farm owners through the Alpaca Association Benelux (AAB). The AAB played no role in the final selection of participating farms nor in the collection, analysis, and interpretation of data, nor in the decision to submit the manuscript for publication. BP acted as an unpaid consultant for AAB.

## Data Availability Statement

The datasets presented in this article are not readily available due to ongoing research projects but are available from the corresponding author on reasonable request. Requests to access the datasets should be directed to Kirsten Proost, kirsten.proost@ugent.be.

## Ethics Statement

Ethical review and approval was not required for the animal study because individual owner consent was obtained for every examination. This study only involves a preventative screening for dental disorders in alpacas. No laboratory animals have been used. Written informed consent was obtained from the owners for the participation of their animals in this study.

## Author Contributions

KP designed the study, collected and analyzed the data, and wrote the manuscript. BP contributed to the study design, data analysis, and revision of the manuscript. LV contributed to the study design, data collection, and revision of the manuscript. All authors contributed to the article and approved the submitted version.

## Funding

This research project was funded by the Department of Surgery and Anesthesiology from the Faculty of Veterinary Medicine of Ghent University. The funding body had no role in the design of the study and data collection, analysis, and interpretation of data and in writing of the manuscript.

## Conflict of Interest

The authors declare that the research was conducted in the absence of any commercial or financial relationships that could be construed as a potential conflict of interest.

## Publisher's Note

All claims expressed in this article are solely those of the authors and do not necessarily represent those of their affiliated organizations, or those of the publisher, the editors and the reviewers. Any product that may be evaluated in this article, or claim that may be made by its manufacturer, is not guaranteed or endorsed by the publisher.

## Abbreviations

CI: Confidence interval
PD: Periodontal disease
ROC: Receiver operating characteristics
Sn: Sensitivity
Sp: Specificity.
